# Honey bee populations of the USA display restrictions in their mtDNA haplotype diversity

**DOI:** 10.3389/fgene.2022.1092121

**Published:** 2023-01-04

**Authors:** Mohamed Alburaki, Shayne Madella, Jillian Lopez, Maria Bouga, Yanping Chen, Dennis vanEngelsdorp

**Affiliations:** ^1^ USDA-ARS Bee Research Laboratory, Beltsville, MD, United States; ^2^ Agricultural University of Athens, Athens, Greece; ^3^ Department of Entomology, University of Maryland, College Park, MD, United States

**Keywords:** *A. mellifera*, genetic diversity, honey bee populations, haplotype, mtDNA, evolutionary lineages, ND2 gene

## Abstract

The genetic diversity of the USA honey bee (*Apis mellifera* L.) populations was examined through a molecular approach using two mitochondrial DNA (mtDNA) markers. A total of 1,063 samples were analyzed for the mtDNA intergenic region located between the cytochrome *c* oxidase I and II (COI-COII) and 401 samples were investigated for the NADH dehydrogenase 2 (ND2) coding gene. The samples represented 45 states, the District of Colombia and two territories of the USA. Nationwide, three maternal evolutionary lineages were identified: the North Mediterranean lineage C (93.79%), the West Mediterranean lineage M (3.2%) and the African lineage A (3.01%). A total of 27 haplotypes were identified, 13 of them (95.11%) were already reported and 14 others (4.87%) were found to be novel haplotypes exclusive to the USA. The number of haplotypes per state/territory ranged between two and eight and the haplotype diversity *H* ranged between 0.236–0.763, with a nationwide haplotype diversity of 0.597. Furthermore, the honey bee populations of the USA were shown to rely heavily (76.64%) on two single haplotypes (C1 = 38.76%, C2j = 37.62%) of the same lineage characterizing *A. m. ligustica* and *A. m. carnica* subspecies, respectively. Molecular-variance parsimony in COI-COII and ND2 confirmed this finding and underlined the central and ancestral position of C2d within the C lineage. Moreover, major haplotypes of *A. m. mellifera* (M3a, M7b, M7c) were recorded in six states (AL, AR, HI, MO, NM and WA). Four classic African haplotypes (A1e, A1v, A4, A4p) were also identified in nine states and Puerto Rico, with higher frequencies in southern states like LA, FL and TX. This data suggests the need to evaluate if a restricted mtDNA haplotype diversity in the US honey bee populations could have negative impacts on the beekeeping sustainability of this country.

## 1 Introduction

The western honey bee species, *A. mellifera *L. is not native to the United States of America. It was first imported in the early 1600s and importations have continued since (Carpenter and Harpur, 2021; Marcelino et al., 2022). However, many indigenous non-*Apis* bee species existed in the country long before the importation of the domestic honey bee to this part of the world. From an ecological and agricultural perspective, all bee species, including non-*Apis* species, solitary bees and honey bees, play a crucial role in pollinating agricultural food crops and wild flowering plants. Nonetheless, the honey bee remains at the forefront of public interest due to its quantifiable economic benefits. Moreover, the flexibility and efficiency when dispatching honey bee colonies to provide pollination services, especially in the USA, are remarkable and unmatched by any other bee species. The direct economic value of the pollination service provided by honey bees is roughly estimated at 17 billion dollars in the USA (Calderone 2012), and some crops and industries, such as almond production, rely entirely on honey bees for pollination. As a non-native species, the honey bee genetic stock in the USA is limited by the import of genetics from the Old World. Healthy genetic diversity within honey bee populations is critical to provide tolerance and resistance to current and future diseases and predators. Honey bees have suffered high losses over the last decade (Kulhanek et al., 2017), emphasizing the need to understand what drives these losses. A lack of genetic diversity has been suggested as one such cause (Panziera et al., 2022). Considering the crucial role honey bees play in agriculture, as many crops depend on them for pollination, it is important to have a current and realistic estimation of the genetic diversity among the honey bee populations of the USA.

Honey bee diversity across the world was initially assessed based on morphometric characteristics like tergite pigmentation, body and wing sizes, pilosity, tomentum width, cubital index and length of the proboscis (Ruttner et al., 1978; Ruttner 1988; Ftayeh et al., 1994; Kauhausen-Keller et al., 1997; Kandemir et al., 2000; Arias et al., 2006; Meixner et al., 2007; Alburaki and Alburaki, 2008). In line with these attributes, four honey bee evolutionary lineages were described; West Mediterranean lineage M), North Mediterranean lineage C), African lineage A) and Oriental lineage O) (Ruttner et al., 1978; Rinderer, 1986; Ruttner, 1988). Despite some discrepancies among recent studies, all four lineages were molecularly confirmed using mitochondrial and microsatellite markers (Smith and Brown, 1988; Smith and Brown, 1990; Cornuet et al., 1991; Smith et al., 1991; Garnery et al., 1992; Garnery et al., 1993; Moritz et al., 1994; Estoup et al., 1995; Garnery et al., 1998a; Garnery et al., 1998b; Meixner et al., 2000; Bouga et al., 2005). Currently, 31 honey bee subspecies have been identified in their natural areas of repartition in Europe, the Middle East, Western Asia and the African continent (Ruttner 1988; Sheppard and Meixner, 2003; Chen et al., 2016).

Mitochondrial DNA (mtDNA) is a widely used tool in phylogeography and genetic studies due to its absence or low recombination, relatively high evolutionary rate, conserved structure and uniparental inheritance (Avise et al., 1987; Harrison 1989; Avise, 2000). This study used mtDNA to examine the haplotype diversity of *Apis mellifera* L. (the honey bee) in the USA. The DraI mtDNA COI-COII (DmCC) test (Smith and Brown, 1990; Cornuet et al., 1991; Garnery et al., 1993) stands as one of the most comprehensive mitochondrial markers and is widely used in honey bee genetics (Hall and Smith, 1991; Arias and Sheppard, 1996; De la Rua et al., 1998; Franck et al., 2000; Palmer et al., 2000; Franck et al., 2001; Abrahamovich et al., 2007; El-Niweiri and Moritz, 2008; Alburaki et al., 2011; Rortais et al., 2011; Loucif-Ayad et al., 2014). The DmCC test consists of conducting a CAPS assay (cleaved amplified polymorphic sequence), also known as PCR-RFLP (restriction fragment length polymorphism), of the mtDNA region, which is located between the cytochrome *c* oxidase subunits I and II. The COI-COII locus is a non-coding region where the restriction patterns reflect the genetic evolution and diversity of *A. mellifera*. In its traditional form, the DmCC test did not require sequencing of the intergenic region as the evolutionary lineages were assigned based on the molecular weight of the amplicons and the haplotypes based on the patterns of the restricted fragments (Garnery et al., 1993). Numerous studies have employed this marker worldwide and hundreds of haplotypes were identified based on the rules mentioned above. Nonetheless, significant discrepancies concerning haplotype naming and identity have arisen among these investigations, further complicating honey bee phylogeographic analyses. In order to reconcile such discrepancies, an *in silico* DmCC test was proposed and tested in a recent study (Madella et al., 2021).

In this study, we conducted a comprehensive genetic assessment of the mtDNA haplotype diversity present in the honey bee populations of the USA. The most successful and widely circulating haplotypes among beekeepers and novel haplotypes of different lineages unique to the honey bee populations of the USA were identified.

## 2 Materials and methods

### 2.1 Honey bee sampling

This study was conducted using samples from the 2019 APHIS National Honey Bee Disease Survey (NHBDS) and three populations (AZ, MS, MD) sampled in 2020 by the USDA-ARS Bee Research Lab in Beltsville. Samples were collected from migratory and stationary apiaries operated by commercial, sideliner and hobbyist beekeepers. One bee was analyzed from each sample. NHBDS samples consisted of ∼150 bees (1/4 cup) from each of 8 colonies in an apiary. Non-NHBDS samples were collected by sampling a single bee per colony from multiple apiaries per state. Both surveys sampled apiaries belonging to different beekeepers and contained eight or more colonies.

### 2.2 DNA extraction

A single leg was dissected from each bee sample and the DNA was extracted in 96-well PCR plates using the Chelex method (Walsh et al., 2013) with slight modifications (Madella et al., 2021). Each leg was individually placed in a plate well and 100 *μ*L of warm (60°C) and constantly stirred 10% Chelex solution (w/v) was added to each plate well. The Chelex solution consisted of 1 g Chelex powder and 10 mL molecular grade water. Prior to sealing the plates, 6 *μ*L of Proteinase K (10 mg/mL) was added to each well. The plates were sealed and placed on a CFX1000 Touch Bio-Rad Thermocycler with the following cycling parameters: 55°C for 1h, 99°C for 15min, 37°C for 1 min, 99°C for 15 min and a final hold at 4°C. DNA extracts were stored at −20°C for further downstream analyses.

### 2.3 Amplification of the mtDNA COI-COII region

#### 2.3.1 DmCC test *in silico*

The intergenic non-coding region of the mtDNA located between the cytochrome oxidase I and II genes was amplified using a new set of validated primers (Madella et al., 2021) and captured the full sequence length of the COI-COII region: COI_Seq-F: ACC​ACC​TCT​AGA​TCA​TTC​ACA​TTT, COII_Seq-R: AGG​ATG​GAA​CTG​TTC​ATG​AAT​GAA. This PCR amplification was conducted in a 14 *μ*L reaction mixture that included 6 *μ*L of Bio-Rad’s Master Mix for PCR (2X), 0.5 *μ*L of each primer (10 *μ*M), 5 *μ*L nuclease-free water and 2 *μ*L of DNA. PCR cycling, conducted on a Bio-Rad C1000 Touch Thermal Cycler (CFX96 Real-Time System), was as follows: 92°C for 3 min, followed by 35 cycles of (92°C for 30 sec, 47°C for 90 sec, 63°C for 2 min), 63°C for 10 min and a final hold at 4°C. Electrophoresis on 1% agarose gel was occasionally conducted on a random set of samples to verify proper amplification of the region prior to Sanger sequencing of the PCR products.

#### 2.3.2 Traditional DmCC test (PCR-RFLP)

The previously and widely used set of primers to traditionally carry out the mtDNA DraI COI-COII test on gels (Garnery et al., 1993; Meixner et al., 2000; Franck et al., 2001; Alburaki et al., 2011; Rortais et al., 2011; Achou M et al., 2015) was used in our study for validation purpose: E2-F: 5′-GGC​AGA​ATA​AGT​GCA​TTG-3′, H2-R: 5′-CAA​TAT​CAT​TGA​TGA​CC-3’. The PCR reaction for this set of primers was carried out in a 26 *μ*L reaction composed of 12.5 *μ*L of Bio-rad’s 2X Master Mix for PCR, 1 *μ*L of each primer (10 *μ*M), 9.5 *μ*L nuclease-free water and 2 *μ*L of DNA. The PCR cycling was similar to the *in silico* test as described above.

### 2.4 Amplification of ND2 gene

The coding gene NADH dehydrogenase 2 (ND2) was amplified on a representative set of samples (n = 401) using the same DNA extracts of the DmCC test. This gene was amplified using the primers described in (Arias and Sheppard, 1996): ND2-F: TGA​TAA​AAG​AAA​TAT​TTT​GA, ND2-R: GAA​TCT​AAT​TAA​TAA​AAA​A. The PCR reaction was carried out as described for the *in silico* DmCC test with the following cycling parameters: 92°C for 3 min, followed by 30 cycles of (92°C for 30 sec, 47°C for 90 sec, 63*°*C for 2 min), 63*°*C for 10 min and a final hold at 4*°*C. Samples were stored at −20*°*C for further analyses.

### 2.5 COI-COII & ND2 sequencing

The PCR products for both COI-COII (n = 1,063) and ND2 (n = 401), which each contained a single amplified band, were purified *via* PCR clean-up procedure and Sanger sequenced from one end (5′end) by Azenta Life Sciences (South Plainfield, NJ, USA). Sequences of novel haplotypes and those exceeding (∼600 bp) were confirmed with a second sequencing conducted from both ends (5′, 3′).

### 2.6 Constitution of COI-COII databases and blasting

In order to achieve 100% accuracy in haplotype identification with available literature and previously published sequences, the intergenic region’s complete sequence must be captured, including the last 62 bp of the tRNA-Leu (3′end) and the first 317 bp of the cytochrome oxidase II (5′end). Unfortunately, a significant number of available COI-COII sequences on NCBI do not meet this criterion and come short at both 5′ and 3′ ends of this region. Our previous study discussed this challenge (Madella et al., 2021). Therefore, among all available COI-COII sequences found on NCBI, a local database exclusively made of verified sequences with complete length was created. This database consisted of 53 worldwide PopSets with 934 full and verified sequences available in the Supplementary Materials. The COI-COII sequences generated in this study were blasted against this database using Geneious Prime software (Megablast Program) with a scoring value of 1 to 2, a linear gap cost and a maximum E-value of 0.05.

### 2.7 Evolutionary lineage & haplotype identification

The honey bee evolutionary lineages were determined based on the genetic structure of the intergenic region detailed in previous studies (Cornuet et al., 1991; Smith et al., 1991; Garnery et al., 1992; Garnery et al., 1993; Alburaki et al., 2011). In order to identify the evolutionary lineages and haplotypes, the DmCC test was conducted *in silico* on all the COI-COII sequences (n = 1,062) following the steps described in (Madella et al., 2021) using Geneious Prime software. Briefly, the sequences were grouped based on the structure and length of the intergenic region, aligned and trimmed at the exact 5′ and 3’ ends of the E2 and H2 primers, annotated and subjected to *in silico Dra*I restriction. The *Dra*I restriction fragments were subsequently revealed on virtual gel with the Lambda DNA/EcoR1 Molecular Marker (ThermoFisher, NY, USA) and other restriction profiles of previously identified haplotypes from prior studies. In order to validate the *in silico* restriction fragment profiles and analyses, the traditional DmCC test was conducted on a representative set of samples from all lineages, including novel haplotypes, which were identified by the *in silico* test. The traditional DmCC test, as described in previous studies (Garnery et al., 1993; Alburaki et al., 2011), is performed *via* a PCR-RFLP on DNA extracts followed by visual identification and comparison of the restricted fragments on polyacrylamide gels.

### 2.8 Naming of novel haplotypes

Genetic studies on honey bees have generated significant datasets, including many partial sequences and unnamed sequences, sequences with multiple names, and distinct sequences assigned the same name. To improve clarity, newly generated data using the DmCC test requires a systematic approach to naming new sequences, offering uniqueness in the haplotypes’ identities and reconciliation between both traditional (PCR-RFLP) and sequencing methods used for haplotype identification. Since the *in silico* DmCC test (Madella et al., 2021) can capture the full length of the tRNALeu–COII region, identify variations within the *Dra*I restriction fragments (virtual gels) and any SNP occurring along this region, we used the following universal rule for naming novel haplotypes: (XD −Y−R−Z ), where X is the alphabetic letter of the lineage (C, M, A), D is a digital number (1 - ∞ ), Y is the full sequence length (bp) between both E2 and H2 primers, R is the number of *Dra*I restricted fragments observed on a virtual gel, and Z is the United Nation Alpha 3 code of the country in which the haplotype originated from (e.g., M5-817-4-USA). This naming convention, if widely adopted, ensures uniqueness in names, avoids discrepancies between research institutions and provides key information related to the characteristic of haplotype and its origin (lineage, digit, sequence length, *Dra*I profile, country of origin). Concerning the *Dra*I profile of the C haplotypes (lineage C), this profile is restricted to 4 fragments only, which led us to drop the last digit from their names: e.g. (C5-571-USA). This nomenclature was only applied to novel haplotypes identified in our study, as we used the accepted names of previously named haplotypes.

### 2.9 Data analysis

Sequence alignment, generation of FASTA files and phylogenetic trees were conducted using Geneious Prime 2022.0.1 (https://www.geneious.com). Geographical mapping of populations was conducted using Tableau Public platform (https://www.tableau.com). Generation of haplotype networks and computation of genetic relationships (haplotype and nucleotide diversity, Tajima’s statistic) were conducted in the R environment (R Core Team, 2022) (v. 2022.07.0) using two main Libraries: “ape” and “pegas” (Paradis, 2010; Paradis and Schliep, 2019). Heatmaps were also generated with R using “pheatmap” and “gplots” Libraries. Haplotype diversity (*H*) is the probability that two randomly sampled alleles are different, while nucleotide diversity (*π*) represents the average number of nucleotide differences per site in pairwise comparisons among DNA sequences (Nei, 1987). In order to compare our data with previous literature, *H* was calculated in two ways. 1) Based on endonuclease fragment variations (haplotypes with no *DraI* restriction pattern differences were considered identical) as proposed in (Nei and Tajima, 1981): $H=\frac{N}{N-1}\left(1-\underset{i}{\sum }X_{i}^{2}\right)$ , where (*H*) is the haplotype diversity, (*N*) is the sample size of the population and $\left(X_{i}\right)$ is the relative haplotype frequency of each haplotype within the same population. 2) Based on DNA sequence variations in which, any single nucleotide change would qualify the sequence as a new haplotype (Nei and Tajima, 1981).

Genetic relationships between haplotypes were computed in R using the molecular-variance parsimony technique. Haplotype networks were estimated under haplotype pairwise differences, which generates the number of mutation steps between haplotypes. ND2 sequences were first aligned using a Cost Matrix of 65% similarity (5.0/4.0) with a “Global alignment with free end gaps” option, then trimmed at both ends to eliminate non-identified nucleotides. The non-coding COI-COII sequences however, were first sorted out according to the genetic structure and length of the intergenic region (Q, P, P0, PQ_x_, P0Q_x_), where x is a tandem repeat of the Q fragment and analyzed following the steps described in a previous study (Madella et al., 2021). All sequences generated by this study were submitted to NCBI and are publicly available.

Neighbor-joining trees were conducted following the genetic model distance of Tamura-Nei (Tamura and Nei, 1993) with no outgroups for 978 sequences of the C lineage (Q fragment) and 369 corresponding sequences of the ND2 gene. Within the C lineage, COI-COII sequences were run with six reference samples from NCBI (MW677216, HQ287900, JQ977702, MH939332, JQ977700, MH939345), which represent each of the C1, C3, C2e, C2d, C2c, C2j haplotypes respectively. The ND2 sequences of the C lineage were analyzed with a set of available reference samples (n = 22)*.* Inter-lineage and intra-C haplotype phylogenetic analyses were conducted on a representative set of ND2 sequences with *Apis cerana* as an outgroup as well as reference sequences of *A. m. mellifera, A. m. macedonica, A. m. ligustica*, *A. m. carnica*, *A. m. sicula*, *A. m. cecropia*, *A. m. pomonella*, *A. m. caucasica, A. m. anatoliaca*, *A. m. meda*, *A. m. syricaca*, *A. m. lamarckii*, *A. m. intermissa*, *A. m. sahariensis*, *A. m. capensis* and *A. m. scutellata* subspecies. These honey bee subspecies represent the four evolutionary lineages known so far (A, M, C, O).

## 3 Results

### 3.1 Sample distribution

**FIGURE 1:**
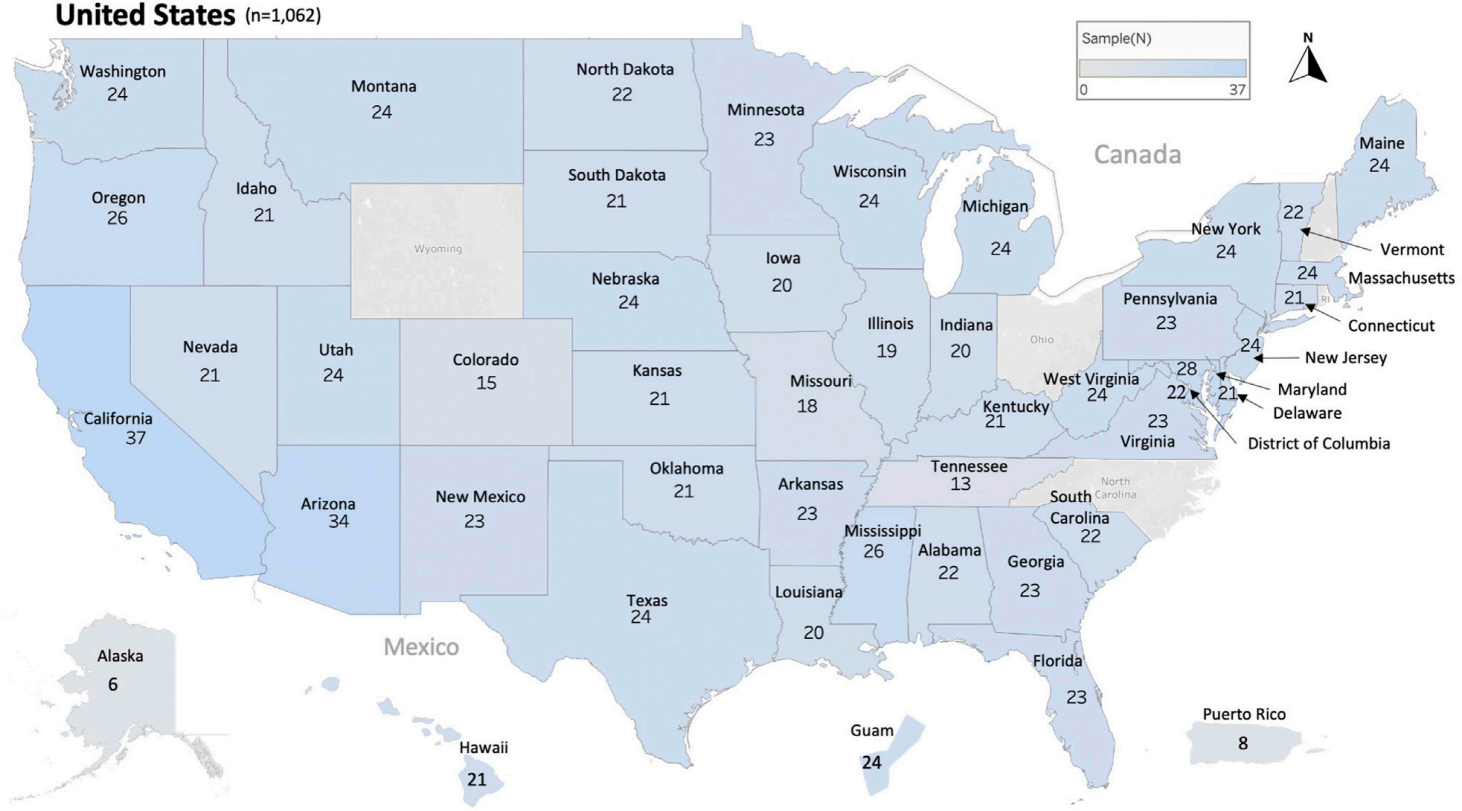
Distribution and number of honey bee samples originated from 45 states, the District of Columbia and two territories of the United States of America USA. Total number of samples nationwide is (n = 1,062). Each sample represents a distinct apiary composed of 8 colonies or more.

In this study, 1,063 samples were analyzed from 45 States of the USA, the District of Columbia (D.C.) and two US territories (Guam and Puerto Rico), Figure 1 and Table 1. In most cases, each sample represents the honey bee operation of a single beekeeper. Honey bee samples from New Hampshire, Rhode Island, Wyoming, Ohio and North Carolina were unavailable. The average number of samples analyzed per state was 22 samples except in relation to Alaska (6), Tennessee (13) and Puerto Rico (8), Figure 1.

**TABLE 1:** COI-COII genetic structure (S), evolutionary lineage (L), sequence length (bp), number of samples (n) and overall percentage of each haplotype nationwide (%). (*) indicates a double band of the same size in the DraI profile. Total one is for samples of previously identified haplotypes and Total two is for novel haplotypes identified in the USA populations.

S	L	Haplotype	DraI profile (bp)	bp	NCBI reference	Blast %	Subspecies/lines	n	%
Q	C	C1	41/47/64/420	572	MW677216	100	*ligustica*	412	38.76
Q	C	C2j	40/47/64/420	571	MH939345	100	*carnica*	400	37.63
Q	C	C2c	40/47/64/420	571	JQ977700	100	*carnica*	58	5.46
Q	C	C2d	40/47/64/420	571	MH939332	100	*carnica*	45	4.23
Q	C	C2i	40/47/64/420	571	MH939339	100	*carnica*	21	1.98
Q	C	C3	40/47/63/421	571	HQ287900	100	Buckfast	42	3.95
PQ	M	M3a	47/65/95/422	629	KX463884	100	*mellifera*	3	0.28
PQ2	M	M7c	47/65*/95/131/422	825	MW677215	100	*mellifera*	1	0.09
PQ2	M	M7b	47/65*/95/131/422	825	MW677214	100	*mellifera*	2	0.19
P0Q	A	A1e	47/108/483	638	MW677198	100	African	12	1.13
P0Q	A	A1v	47/108/483	638	MW677201	100	African	2	0.19
P0Q2	A	A4	47/108/192/483	830	MW939614	100	African	3	0.28
P0Q2	A	A4p	47/109/192/483	831	MW677211	100	African	10	0.94
**Total 1**								**1,011**	**95.11**
**Novel Haplotype**									
Q	C	C1-571-USA	40/47/63/421	571	HQ2287800	99.9	*carnica*	7	0.66
Q	C	C2-570-USA	40/47/64/419	570	JQ977700	99.9	*carnica*	2	0.19
Q	C	C3-567-USA	40/47/60/420	567	MH939332	99.6	*carnica*	1	0.09
Q	C	C4-571-USA	40/47/64/420	571	MW939587	100	*carnica*	2	0.19
Q	C	C5-571-USA	40/47/64/420	571	MW939577	99.9	*carnica*	1	0.09
Q	C	C6-571-USA	40/47/64/420	571	MH939345	99.9	*carnica*	1	0.09
Q	C	C7-570-USA	40/47/63/420	570	MW677221	100	*carnica*	5	0.47
PQ2	M	M1-825-5-USA	65*/131/142/422	825	KX463897	99.9	*mellifera*	8	0.75
PQ2	M	M5-817-4-USA	47/96/192/482	817	MG592306	99.3	*mellifera*	1	0.09
PQ2	M	M4-817-6-USA	47/65*/95/123/422	817	MW677214	99.5	*mellifera*	3	0.28
PQ3	M	M3-1023-6-USA	65/66/131/132/142/422	1,023	MW939594	99.9	*mellifera*	3	0.28
PQ3	M	M2-1021-7-USA	47/65*/95/131*/422	1,021	KX463940	99.9	*mellifera*	13	1.22
P0Q2	A	A1-837-6-USA	47/67*/107/129/420	837	EU785975	99.6	African	2	0.19
P0Q2	A	A2-829-4-USA	47/107/192/483	829	MW939597	100	African	3	0.28
**Total 2**								**52**	**4.87**
**Grand Total**								**1,063**	**100**

### 3.2 COI-COII and ND2 sequences

A total of 1,063 sequences for the intergenic COI-COII region and 401 sequences for the ND2 coding gene were obtained, Table 1. All sequences were deposited at the National Center for Biotechnology Information (NCBI) GeneBank with the following accession numbers: 1) ND2 sequences (OM219207-OM219607), (OM219608-OM219613), (OM219614-OM219625), 2) COI-COII sequences (OM219608 - OM219613), (OM219614 - OM219625).

### 3.3 Honey bee evolutionary lineage

**FIGURE 2:**
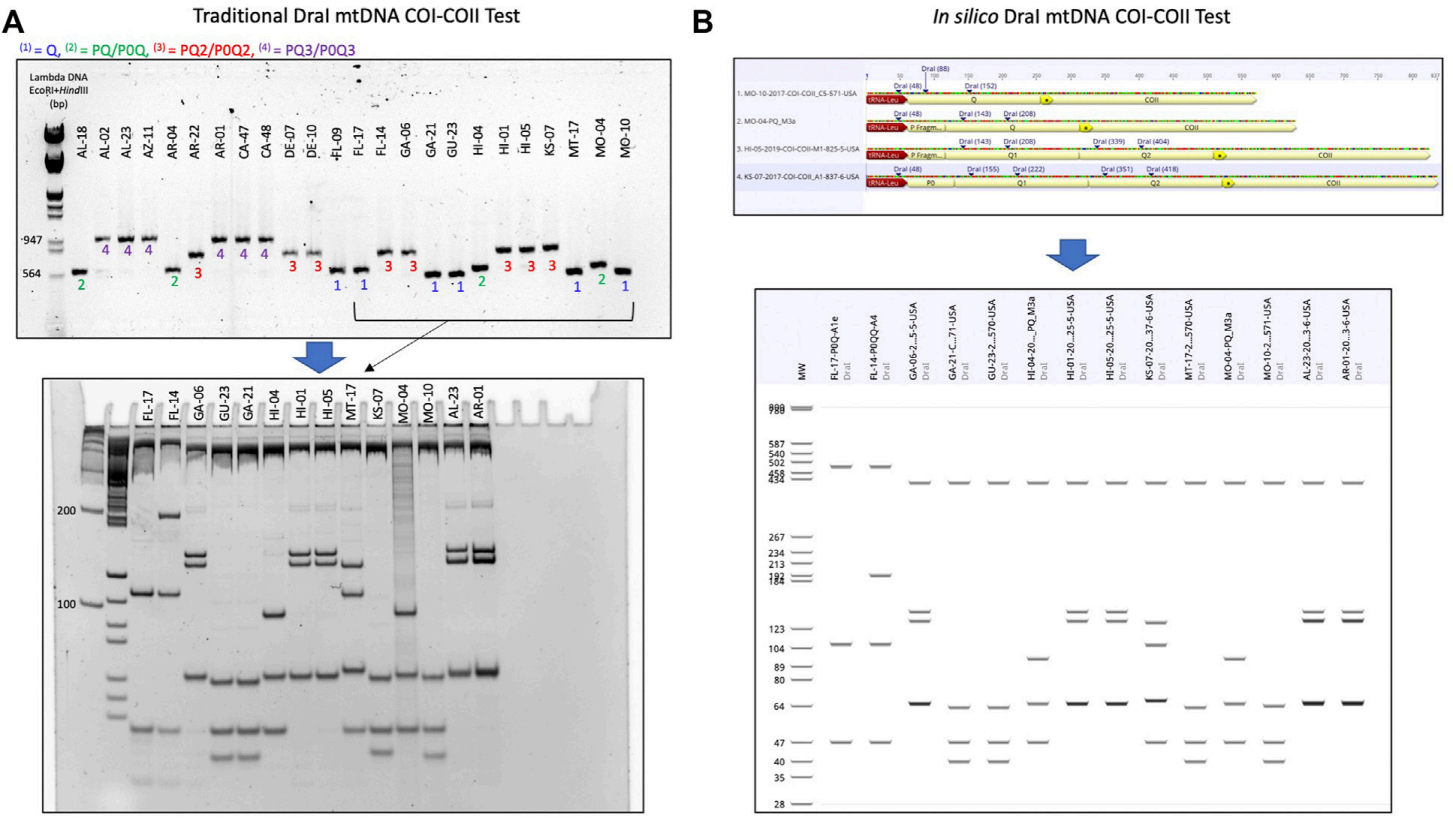
Procedure detailing the transition from the traditional DraI mtDNA COI-COII (DmCC) test to the *in silico* DmCC test. **(A)** Lineage identification based on the mtDNA COI-COII length revealed on 1.5% agarose gel, DraI enzymatic restriction of the PCR products and visual haplotype identification on 7.5% polyacrylamide gel. **(B)** Determination of COI-COII genetic structure (Q, PQ, PQ2, P0Q2) by sequence alignment and annotation. *In silico* DraI restriction of the sequences and haplotype identification on a virtual gel. The same samples shown in **(A)** were run in **(B)**.

**FIGURE 3:**
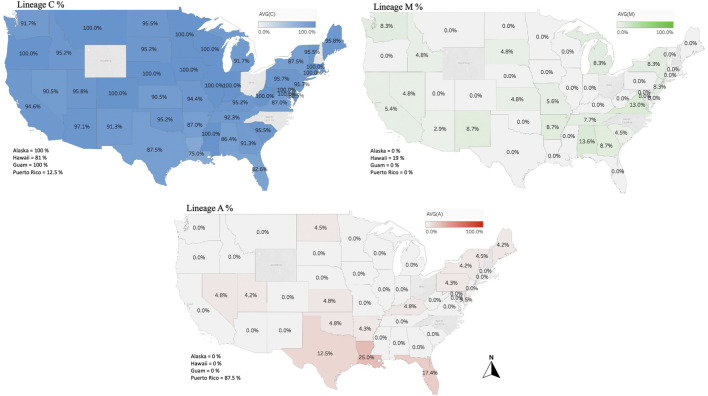
Percentages of the three identified maternal lineages (North Mediterranean C, West Mediterranean M and African lineage A) in the US honey bee populations computed and displayed by states and territories. Samples from the States of OH, NC, NH, WY and RI were not available.

The genetic structure of the mtDNA COI-COII intergenic region of the specified US honey bee populations showed six different polymorphisms: Q, PQ, P0Q, PQ2, PQ3, P0Q2, Table 1 and Figure 2. Furthermore, three evolutionary lineages were identified: 1) North Mediterranean lineage C, 2) West Mediterranean lineage M and 3) the African lineage A, with the C lineage dominating, Table 1 and Figure 3. In total, 93.79% of the samples belonged to the C lineage, 3.2% to the M lineage and 3.01% to the African lineage, Table 1. The M lineage was found in 19 States, while the African lineage was identified in 16 states. Higher African lineage frequencies were found in southern states such as LA (25%), FL (17.4%), TX (12.5%) and the territory of Puerto Rico (87.5%), Figure 3. The last percentage should be carefully considered in light of a restricted number of analyzed samples from this population (n = 8) compared to other states (n = 22), Table 2 and Figure 3.

**TABLE 2:** Haplotype identity and occurrence on a state-by-state basis. Sample size (n), total number of haplotypes found in each population (x). Novel haplotypes found in the USA were named according to the nomenclature detailed in this study.

Sates	Code	N	x	Haplotype ID	Novel haplotype
Alabama	AL	22	7	C1, C2j, C2c, C2d, C3, M3a	M3-1023-6-USA
Alaska	AK	6	3	C1, C2j, C2d	-
Arizona	AZ	34	4	C1, C2j, C2d	M2-1021-7-USA
Arkansas	AR	23	8	C1, C2j, C2c, C2d, C3, M7c, A1v	M3-1023-6-USA
California	CA	37	4	C1, C2j, C2d	M2-1021-7-USA
Colorado	CO	15	3	C1, C2j, C2c	-
Connecticut	CT	21	3	C1, C2j, C2d	-
Delaware	DE	21	6	C1, C2j, C2c, C3, A4, A4p	-
District of Columbia	DC	22	5	C1, C2j, C2c, C2d, C3	-
Florida	FL	23	7	C1, C2j, C2c, C2d, C3, A1e, A4	-
Georgia	GA	23	6	C1, C2j, C2c	C1-571-USA, M1-825-5-USA, M2-1021-7-USA
Guam	GU	25	4	C1, C2j, C2i	C7-570-USA
Hawaii	HI	21	5	C1, C2j, M3a	M2-1021-7-USA, M1-825-5-USA
Idaho	ID	21	4	C1, C2j, C3	M2-1021-7-USA
Illinois	IL	19	6	C1, C2j, C2c, C2d, C3	C4-571-USA
Indiana	IN	20	4	C1, C2j, C2d, C3	-
Iowa	IA	20	4	C1, C2j, C2c, C3	-
Kansas	KS	21	4	C1, C2j	M2-1021-7-USA, A1-837-6-USA
Kentucky	KY	21	4	C1, C2j, C2c, A1e	-
Louisiana	LA	20	7	C1, C2j, C2c, C2d, C3, A1e	A2-829-4-USA
Maine	ME	24	5	C1, C2j, C2c, C2d, A4p	-
Maryland	MD	28	4	C1, C2j, C2c, C2d	-
Massachusetts	MA	24	5	C1, C2j, C2c, C2d, C3	-
Michigan	MI	24	7	C1, C2j, C2c, C2d, C3	M2-1021-7-USA, C1-571-USA
Minnesota	MN	23	5	C1, C2j, C2c, C2d	C1-571-USA
Mississippi	MS	26	2	C1, C2j	-
Missouri	MO	18	6	C1, C2j, C2d, C3, M3a	C5-571-USA
Montana	MT	24	6	C1, C2j, C2c, C2d	C1-571-USA, C7-570-USA
Nebraska	NE	24	4	C1, C2j, C2c, C2d	-
Nevada	NV	21	5	C1, C2j, C2c, A4p	M5-817-4-USA
New Jersey	NJ	24	5	C1, C2j, C2d	M1-825-5-USA, M4-817-6-USA
New Mexico	NM	23	7	C1, C2j, C2c, C2d, C2i, M7b	M1-825-5-USA
New York	NY	24	5	C1, C2j, A4p	M1-825-5-USA, M2-1021-7-USA
North Dakota	ND	22	5	C1, C2j, C2c	A2-829-4-USA, C6-571-USA
Oklahoma	OK	21	5	C1, C2j, C3, A1e	C4-571-USA
Oregon	OR	26	5	C1, C2j, C2d	C7-570-USA, C3-567-USA
Pennsylvania	PA	23	4	C1, C2j, C3, A4	-
Puerto Rico	PR	8	3	C2j, A4p	A1-837-6-USA
South Carolina	SC	22	6	C1, C2j, C2d, C3	M2-1021-7-USA, C2-570-USA
South Dakota	SD	21	3	C1, C2j	M2-1021-7-USA
Tennessee	TN	13	5	C1, C2j, C2c, C3	M4-817-6-USA
Texas	TX	24	4	C1, C2j, C2d, A1e	-
Utah	UT	24	4	C1, C2j, C2d, A1v	-
Vermont	VT	22	6	C1, C2j, C2c, C2d, C3	A2-829-4-USA
Virginia	VA	23	6	C1, C2j, C2c, C3	M1-825-5-USA, M4-817-6-USA
Washington	WA	24	6	C1, C2j, C2d, C3, M7b	M2-1021-7-USA
West Virginia	WV	24	5	C1, C2j, C2c, C2d, C3	-
Wisconsin	WI	24	5	C1, C2j, C2c, C2d	C1-571-USA

### 3.4 COI-COII haplotypes

**FIGURE 4:**
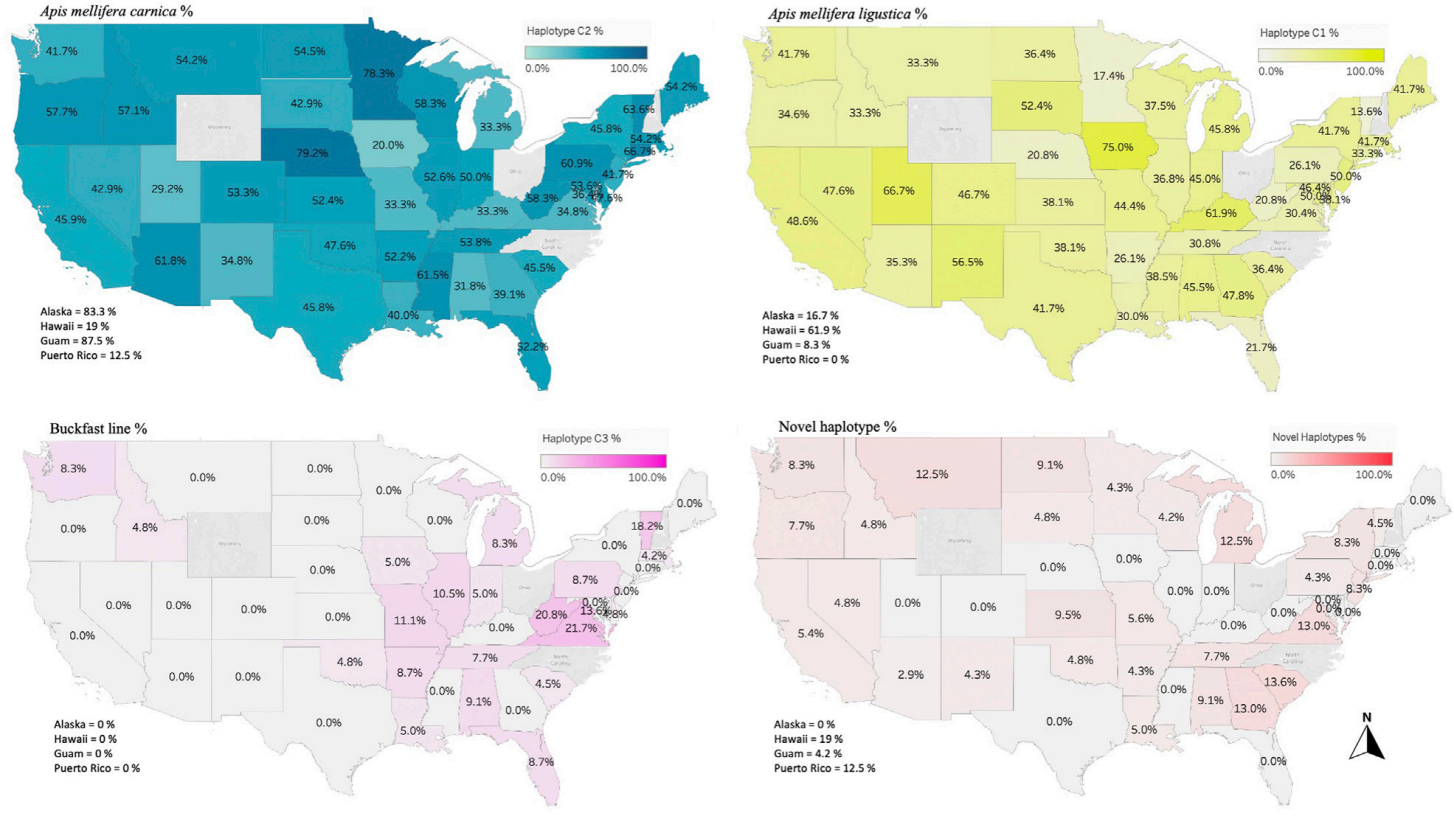
Major three haplotypes found in the honey bee populations of the USA. Haplotype C1 characterizes the honey bee subspecies *A. m. ligustica* (Italian honey bee), C2 haplotype for *A. m. carnica* (Carniolan honey bee) and C3 represents the Buckfast line. Novel haplotypes found in this study are also provided in percentage of the total analyzed samples per state/territory.

Overall, 27 haplotypes were identified; 13 (95.11%) had already been reported and 14 (4.87%) others were deemed to be novel haplotypes, Table 1. By far, the two predominant haplotypes found in the USA are C1 and C2j, representing (38.76%) and (37.62%) of the total sample size, respectively (Table 1). The C1 and C2j haplotypes characterize the *A. m. ligustica* and *A. m. carnica* subspecies, respectively Figure 4 (Alburaki et al., 2011; Bouga et al., 2011). Haplotype C2i was exclusively found in the isolated island of Guam at a very high rate (87.5%), excluding a single occurrence identified in NM, Table 2. Haplotype C3 which characterizes the Buckfast line (Alburaki et al., 2011; Bouga et al., 2011) was recorded 42 times in an overall percentage of 3.95%, Table 1 and Figure 4. Three typical M haplotypes of *A.m. mellifera* subspecies (M3a, M7b, M7c) (Rortais et al., 2011; Bertrand et al., 2015) were found in solitary instances in AL, AR, HI, MO, NM and WA. Meanwhile, commonly known haplotypes of the African lineage (A1e, A1v, A4, A4p) were recorded in 11 states and a territory (Puerto Rico), with higher frequencies in Southeastern states (LA, FL, TX), Figure 3 and Table 2.

### 3.5 Novel haplotypes

Seven of the 14 novel haplotypes documented in this study belong to the C lineage, five to the M lineage and two to the A lineage, Table 1. Additionally, three novel haplotypes (C4-571-USA, C7-570-USA, A2-829-4-USA) blasted at 100% with orphan sequences deposited at NCBI with no track records or haplotype identity to reference, Table 1. The five novel haplotypes of the M lineage were identified at low frequencies (1–13) and percentages (0.09%–1.22%) in AL, AZ, AR, CA, HI, ID, KY, MI, NV, NJ, NM, NY, SC, SD, TN, VA and WA, (Table 1 & 2). The most frequently identified novel haplotype (M2-1021-7-USA) was found in 13 apiaries in 11 different States (AZ, CA, GA, HI, ID, KS, MI, NY, SC, SD, WA), signaling significant circulation of this haplotype across the USA, Table 1 & 2. The two novel African haplotypes (A1-837-6-USA, A2-829-4-USA) were shown to exhibit the (P0) fragment of 68bp directly after the tRNA-Leu region with a single replication of the (Q) sequence. Both haplotypes were identified in LA, ND, PR, and VT, Tables 1 & 2.

### 3.6 Haplotype genetic relationship

**FIGURE 5:**
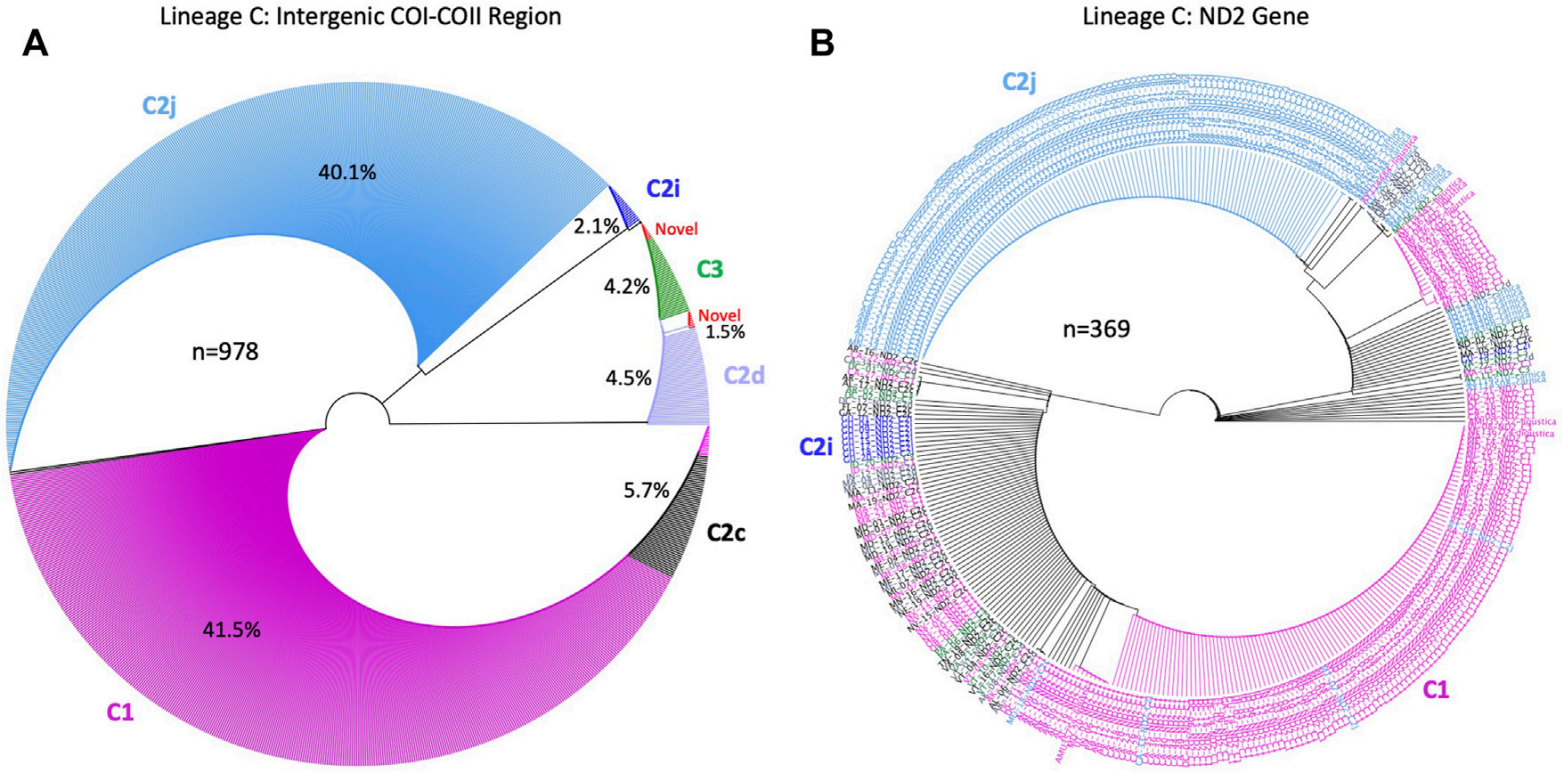
Phylogenetic analysis conducted on sequences of the C lineage in two mtDNA genetic regions: **(A)**: COI–COII intergenic region and **(B)**: NADH dehydrogenase 2 (ND2) coding gene (n) is the number of sequences run in each phylogenetic tree, contribution of each COI-COII haplotypes is given in (%). ND2 sequences in **(B)** were labeled with their respective COI-COII haplotypes identified in **(A)**. Analyses were computed with available NCBI sequences of the C lineage for both genes.

**FIGURE 6:**
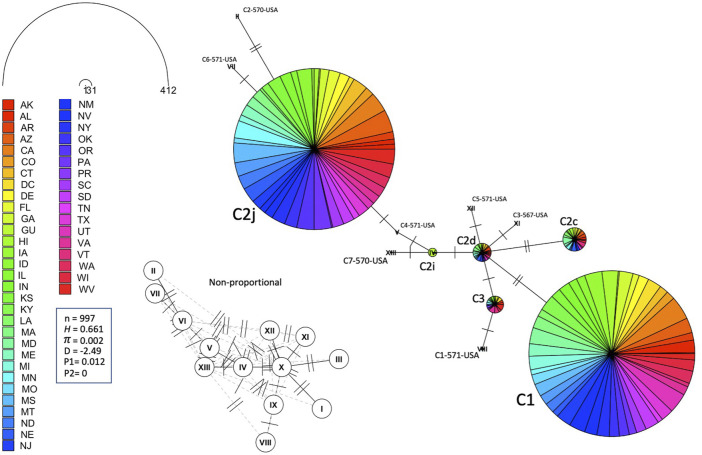
Haplotype network of the mtDNA COI-COII region for samples of Lineage C represented by states. Number of samples (n), haplotype diversity (*H*), nucleotide diversity (*π*), Tajima’s statistic (D), Tajima’s *p*-values under normal (P_1_) and beta (P_2_) distributions. Circle size is relative to the number of haplotype copies in the dataset. Perpendicular lines on branches represent SNP events. Non-proportional subfigure shows haplotype secondary network (dotted line) without haplotype copy representation. C2j and C1 are the two major haplotypes (76.64%) identified in the USA populations.

The number of haplotypes per state ranged between two to eight, with a national average of (4.9 ± 0.2) haplotypes, Table 2. The national average for haplotype diversity (*H*) was (0.597) and it ranged between (0.236–0.763) per state (Supplementary Table S1), while the lowest *H* value was found in GU (0.236) and the highest *H* was recorded in VA (0.763). No corrected *H* was calculated as the studied species is not native to the USA. The nucleotide diversity (*π*) within samples of the C lineage was (0.002) with (*H =* 0.661) and there was an overwhelming frequency of both C1 and C2j haplotypes (Figures 5, 6), confirming the *in silico* DmCC results shown in Table 1. Tajima’s statistic (D = −2.49, *p* < 0.01) indicates a deviation from neutrality and highlights evidence of strong selection activity, Figure 6.

**FIGURE 7:**
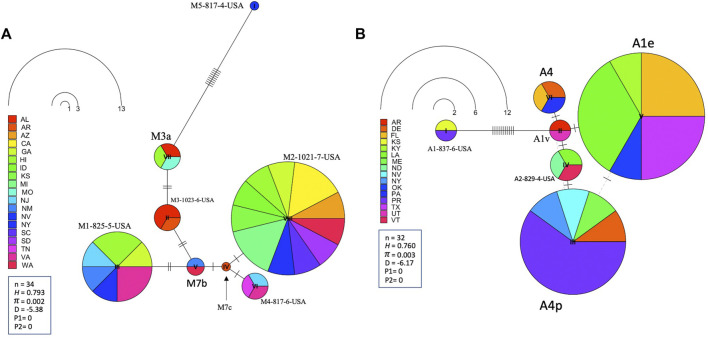
State-by-State representation of the COI-COII haplotype network of both M and A lineages; **(A)** and **(B)** respectively. Number of samples (n), haplotype diversity (*H*), nucleotide diversity (*π*), Tajima’s statistic (*D*), Tajima’s *p*-values under normal (P_1_) and beta (P_2_) distributions. Circle size is relative to the number of haplotype copies present in the dataset. Perpendicular lines on branches represent nucleotide variations.

Leading on from this, the COI-COII haplotype network suggests that C2d is the ancestral haplotype from which other haplotypes of the C lineage have diverged through (≥1) SNPs, Figure 6. Meanwhile, the M haplotype network shows higher frequencies of novel haplotypes in US populations compared to previously identified M haplotypes found in the native range of the M lineage, Figure 7A. The novel M haplotype (M5-817-4-USA) showed significant deviation from other M haplotypes (Figure 7A), similar to the novel A haplotype (A1-837-6-USA), Figure 7B. In contrast to the M lineage, previously identified haplotypes of the A lineage were pervasive in US populations as compared to their A lineage novel counterparts, Figure 7B.

**FIGURE 8:**
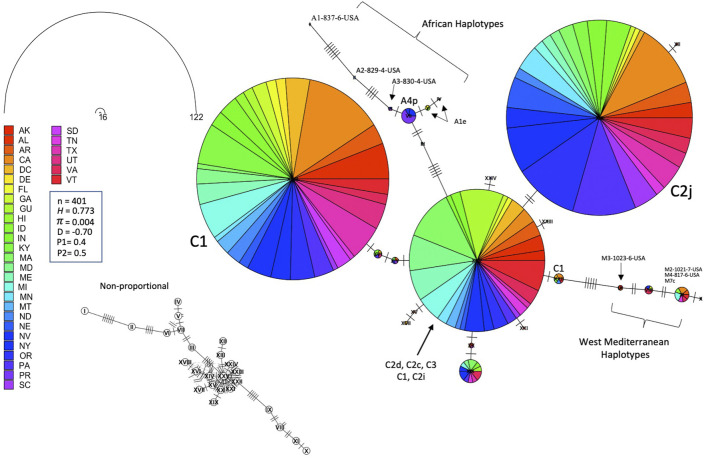
Haplotype network of the ND2 gene for samples of all COI-COII lineages displayed by states. Number of samples (n), haplotype diversity (*H*), nucleotide diversity (*π*), Tajima’s statistic (*D*), Tajima’s *p*-values under normal (P_1_) and beta (P_2_) distributions. Circle size is relative to the number of haplotype copies present in the dataset. Nucleic changes are represented by perpendicular lines on branches. Non-proportional subfigure shows the haplotype secondary network (dotted line) without haplotype copy representation. Central ND2 haplotype is an admixture of multiple COI-COII haplotypes.

**FIGURE 9:**
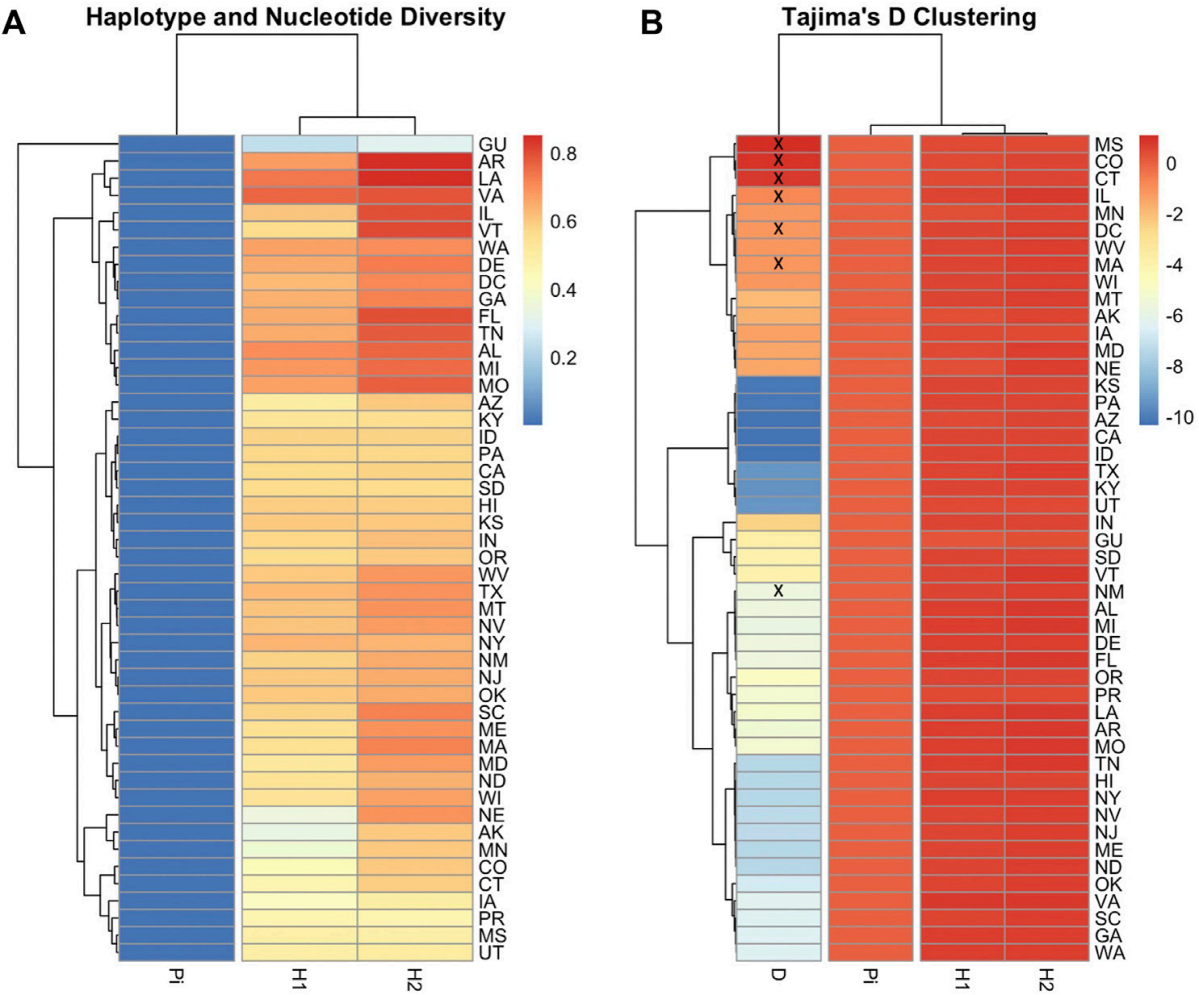
Heatmaps illustrating COI-COII gene diversity per population. Nucleotide diversity (Pi), Haplotype diversity calculated based on endonuclease fragment variations (*H*_*1*_) (Nei and Tajima 1981), Haplotype diversity computed on DNA sequences (*H*_*2*_), Tajima’s statistic (*D*). **(A)**: Genetic relationship among populations based on gene diversity parameters (*H*_*1*_, *H*_*2*_, Pi). **(B)**: Population clusters based on Tajima’s D values. Population with non-significant *D* values (*p* > 0.05) were crossed by (x). Full range of data is available in Supplementary Table S1.

The ND2 sequences followed the trend of the COI-COII network but they exhibited slightly higher haplotype (*H* = 0.773) and nucleotide diversities (*π* = 0.004), which was due to the difference in the nature and rate of evolution between the two genetic regions, Figure 8. This network classified the sequences into three lineages and confirmed the overwhelming findings of the COI-COII region, particularly the significant predominance of both C1 and C2j haplotypes. Figure 8. Major ND2 haplotype groups were labeled with their respective COI-COII identities. Both haplotype diversities (*H*_*1*_, *H*_*2*_) followed similar trends, with *H*_*1*_ being more conservative, Figure 9. The populations with the highest (*H*) were AR, LA, VA, IL and VT. In contrast the population with the lowest *H* value was GU. Tajima’s D indicates that most US populations do not adhere to neutrality and that bottlenecking or high levels of selection pressure are likely occurring among these populations.

### 3.7 Phylogenetic analyses

The unrooted genetic tree of the COI-COII sequences of the C lineage showed four major clusters representing haplotypes C1, C2j, C2d, and C2i, Figure 5A. The novel haplotypes uncovered in this study clustered within the C2d branch along with haplotype C3. Nevertheless, haplotype C2c clustered within the C1 group showing more genetic proximity with *A. m. ligustica* than *A. m. carnica*, Figure 5A. The ND2 coding region demonstrated relatively similar evolution patterns found in the COI-COII region, particularly for C1 and C2j haplotypes, while C2d and C3 did not follow a clear pattern, Figure 5B. In subfigure 5b, ND2 sequences were labeled with the names of their respective COI-COII haplotype. Meanwhile, the alignment of the COI-COII and ND2 sequences provides visual evidence of the actual polymorphisms present among these haplotypes, Supplementary Figure S1. The uniqueness of the C1 haplotype is reflected by a single insertion in its early Q fragment, which cannot be found anywhere else in the C lineage, as well as a substitution in its COII coding region that is shared with C2c and the novel haplotype (C2-570-USA), Supplementary Figure S1.

**FIGURE 10:**
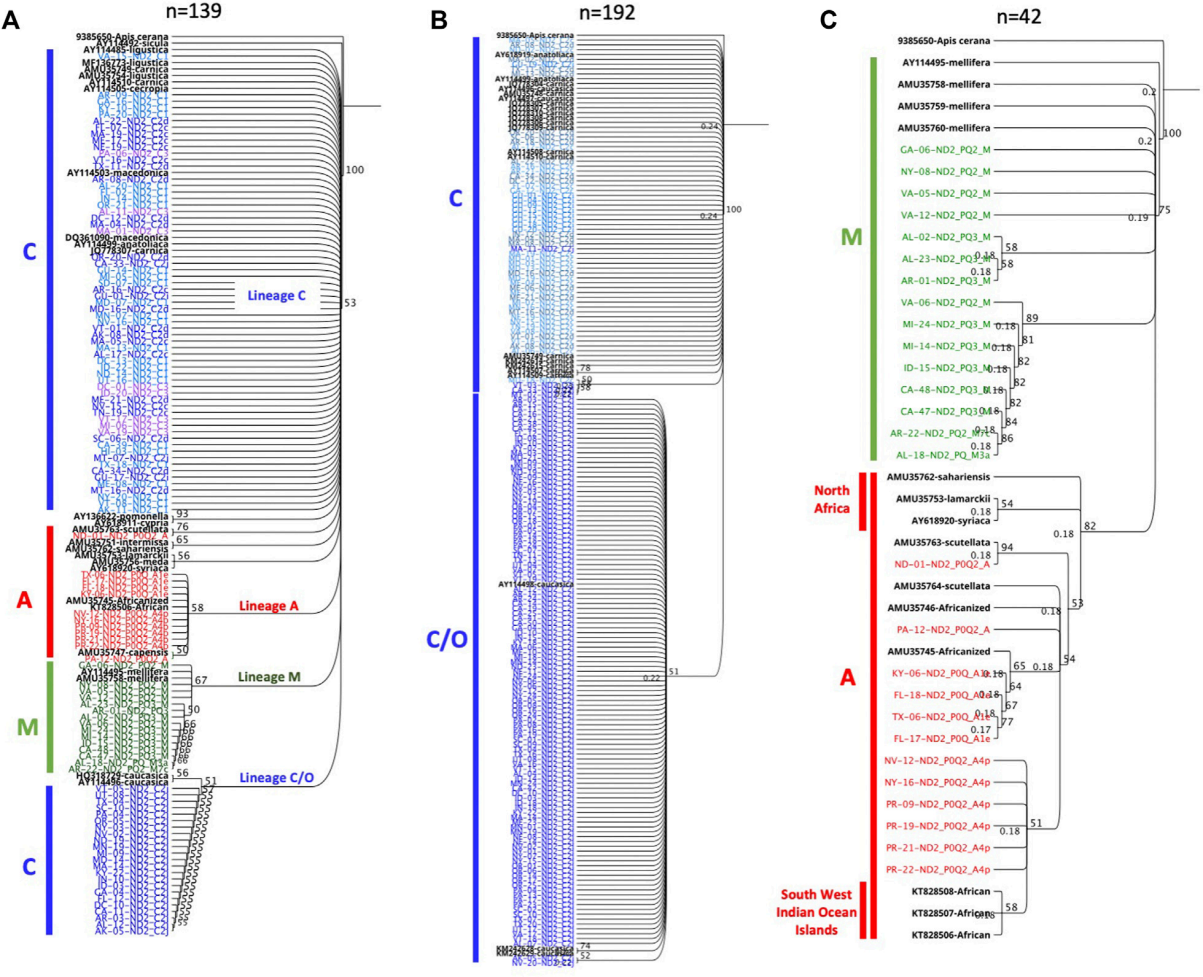
Phylogenetic analyses of ND2 coding gene conducted on representative set of samples from three mtDNA COI-COII lineages (C, M, A). Sequences in black font are NCBI reference samples of major honey bee subspecies. Trees are rooted with *Apis cerana* as an outgroup species and numbers beside the node show bootstrap support values. **(A)** represents inter-lineage analysis (bootstrap = 53). **(B)** intra-C haplotype analysis. **(C)** Samples of lineages M (green) and A (red) discriminated by a bootstrap value of 75.

Multiple deletions and SNPs are observed in the novel haplotypes’ sequences when compared to Old World haplotypes Supplementary Figure S1. Both haplotypes C1 and C2j retained a clear contrast in their ND2 sequences compared to other C haplotypes with a substitution at 40 and 250 bp, respectively, Supplementary Figure S1. This divergence led to their clear distinction (Figure 5) compared to other C haplotypes. The phylogenetic tree rooted with *A. cerana* revealed three clusters representing the three evolutionary lineages (C, M, A) with a bootstrap value of 53, Figure 10A. Moreover, the analysis of intra-C haplotypes (excluding C1) confirmed the presence of a clear distinction with regards to C2j, differentiating it from other C2 sub-haplotypes in which C2j clustered with reference samples of *caucasica* subspecies (morphometrical lineage O) (Ruttner, 1988) (Figure 10B). Samples of M and A lineages in the USA clustered in two independent groups with reference samples of *A. m. mellifera* and other African subspecies (*A. m. sahariensis*, *A. m. lamarckii*, *A. m. syriaca*, *A. m. scutellata*) with a bootstrap value of 75, Figure 10C.

## 4 Discussion

Our data shows that the managed honey bee populations of the USA (n = 1,063) belong to three known honey bee evolutionary lineages (C, M, A) with an overwhelming reliance (93.79%) on the North Mediterranean C lineage. The remaining samples belonged to the West Mediterranean M lineage (3.20%) and the African A lineage (3.01%), Table 1. A previous study conducted on feral honey bee colonies (n = 422) sampled from nine states in the USA found that 77% of the samples belonged to the C lineage, 22% to the M lineage and 1% to the A lineage (Schiff and Sheppard, 1993). In a more recent study conducted on unmanaged colonies (n = 247) from 12 states, 83% of the samples belonged to the C lineage, 7% to the M lineage, while 9% were attributed to the Oriental lineage O) (Magnus et al., 2014). As the Oriental lineage cannot be discerned with the DmCC test, we verified the aforementioned study’s sequences, which revealed that the O lineage should actually be characterized as the African lineage A) (Z haplotypes of *A. m. syriaca*). This issue was discussed in a previous study (Kandemir et al., 2006) and previous COI-COII mtDNA O haplotypes were reclassified accordingly as Z haplotypes of the African lineage A (Alburaki et al., 2011). Another recent study indicated that 100% of the examined managed colonies (n = 11) in PA were of the C lineage (Rangel et al., 2020). Unmanaged colonies, as well as feral swarms, initially originated from the managed stock. Although it is expected for wild populations to follow a more neutral selection path compared to managed counterparts, their diversity often mirrors the managed populations. Taking into consideration our data and previous studies conducted on feral and managed colonies (Schiff et al., 1994; Delaney et al., 2009), it is clear that lineage C is by far the most dominant lineage found in the honey bee populations of the USA, with only traces of M and A lineages.

### 4.1 The North Mediterranean lineage C

This lineage originates from the North Mediterranean region of Southeast Europe. It includes several well-defined subspecies, such as *A. m. ligustica*, *A. m. carnica, A. m. macedonica, A. m. cecropia, and A. m. cypria*. The first two examples are this lineage’s most historically imported and common subspecies (Ruttner 1988; Ilyasov et al., 2020). In our samples, the C lineage is represented by two major haplotypes (C1, C2_x_), which characterize *A. m. ligustica* and *A. m. carnica* subspecies, respectively (Pinto et al., 2014). The Carniolan bee, *A. m. carnica*, was the most abundant subspecies among the USA populations, indicating significant circulation of Carniolan queen stocks, represented by four haplotypes (C2j, C2c, C2d, C2i), with low frequencies for the last three, Table 1. Haplotype diversity among European populations differs starkly from the US populations. In their native range of repartition (Central and Southeastern Europe), a recent phylogeographic study (n = 564) that used the same COI-COII marker identified a total of 13 C2 native haplotypes (C2d, C2c, C2b, C2e, C2i, C2j, C2q, C2u, C2v, C2w, C2aa, C2ab, C2ag) as well as highly common C2d (38%) and C2c (23.8%) haplotypes, with a haplotype diversity *H*) of 0.756 (Munoz and de la Rua, 2021). In US populations, the overwhelming reliance appeared to be on the C2j haplotype (37.63%), while C2d (5.46%) and C2c (4.23%) were much less frequent and only displayed a haplotype diversity (*H*) of 0.542 within the C lineage. As a predominant lineage of the USA, to a great extent, the low *H* within the C lineage mirrors the national *H* (0.597), which should be given careful attention. Interestingly, our study confirms the ancestral position of C2d, which was uncovered in Munoz and De La Rua, 2021, from which C2i deviated by a single mutation, Figure 6. Moreover, Three out of seven novel C haplotypes found in the USA’s populations were derived from the ancestral C2d haplotype by just one or two mutations. Despite its overall low percentage in the USA, C2d is widely distributed and was identified in 28 states, suggesting that a genetic shift towards C2j occurred over time in accordance with natural or human selection. Human selection pressure is more probable in this context as most US populations rejected Tajima’s assumption of “neutral evolution” except in MS, CO, CT, IL, DC, MA and NM populations, Figure 9 and Supplementary Table S1. Further genetic investigation is needed of the C2j haplotype within the USA, which constantly clustered with reference samples of *A. m. caucasica* (Russian bees) in the ND2 phylogenetic analyses, Figure 10.

Italian queens (*A. m. ligustica*)*,* characterized by the C1 haplotype, were the second most widely circulated queens among beekeepers in the USA (38.76%). Within a sample size of 412 C1 haplotypes spread across extensive geographical and climatic ranges, no COI-COII polymorphisms were found in this subspecies. Regarding the C2i haplotype, the absence of queen importation from Guam to the mainland US states could explain its exclusiveness on the isolated Island. All C1 and C2_x_ haplotypes were identified in the Old World (Europe) in their native geographical ranges (Bouga et al., 2011).

Tracking the geographical location and occurrence rate of the rare and novel mtDNA haplotypes offers a powerful tool that could shed even more light on beekeeping and queen circulations nationwide. For instance, our data shows that honey bee queens carrying the most common novel haplotype (C1-571-USA) were found in sampled operations from four states (GA, MN, MT, WI), followed by the second most common novel haplotype (C7-570-USA), which was found in two States (MT, OR) and the isolated island of Guam. All the other novel haplotypes (C2-570-USA, C3-567-USA, C4-571-USA, C5-571-USA, and C6-571-USA) that were found in SC, OR, OK, MO, and ND respectively, have so far remained state-specific. None of these particular novel haplotypes identified in our study were reported elsewhere, including in the native geographical range of the C lineage, which makes them unique and singles them out as valuable material to further our understanding of the honey bee evolution and beekeeping practices in the USA. The most common and circulating C haplotypes in the USA were those haplotypes that are well documented and broadly known in Europe, while novel ones remained rare and state-restricted for some.

### 4.2 The West Mediterranean lineage M

This lineage has been widely studied and comprises numerous documented haplotypes that characterize two subspecies; *A. m. mellifera* and *A. m. iberiensis* (Rortais et al., 2011; Chávez-Galarza et al., 2017; Henriques et al., 2018). Within the US populations, only 3.2% (n = 34) of the queen bees originated from this lineage which carries three of the most predominant and well-known M haplotypes (M3a, M7c, M7b). In its natural areas of repartition (West Europe), at least 91 haplotypes were described in this lineage (Franck et al., 1998; Rortais et al., 2011), while only three of them (M haplotypes) were identified in the USA. This distinction suggests that queens/bees of this lineage were not imported or incorporated into US breeding programs at the same rate as the C lineage. This low M haplotype integration in US beekeeping practices is surprising considering the lineage’s high genetic diversity and multiple identified ecotypes (Louveaux, 1973). In addition to the three haplotypes mentioned above, five novel M lineage haplotypes were identified, Table 1. These novel haplotypes were identified 28 times within 22 states, with the most abundant (M2-1021-7-USA) found in (AZ, CA, GA, HI, ID, KS, MI, NY, SC, SD and WA). The low number of M haplotypes samples (n = 34) found in the USA undermines the possibility of a comprehensive phylogenetic analysis. Nonetheless, it is safe to conclude that novel M haplotypes, which may have developed local adaptations, are used more widely by beekeepers than the traditional M haplotypes originating in Europe, Figure 7A. Among the lineages in the USA, the M lineage exhibited the highest *H* (0.720) Supplementary Table S1, as evidenced by eight identified haplotypes in the USA populations from a relatively small sample size (n = 32). This *H* level is clearly healthier than what was found among USA’s C lineage and it is closer to the higher end (0.324–0.771) of the M (*H*) that was documented in a previous study (n = 219), which was conducted within the lineage’s native range in France and Spain (Franck et al., 1998).

### 4.3 The African lineage A

This lineage is native to the African continent and is considered the most genetically diverse lineage comprising over 13 subspecies, such as *A. m. lamarckii*, *A. m*. *intermissa*, *A. m*. *syriaca*, *A. m*. *scutellata*, *A. m*. *capen, A. m*. *siciliana* and *A. m*. *unicolor* (Franck et al., 2001; Alburaki et al., 2011; Henriques et al., 2022). Unfortunately, only a few subspecies of this lineage were characterized by specific haplotypes, which renders the exact determination of a subspecies by molecular means challenging. Africanized honey bees are the results of crossbreeding between *A. m. scutellata* with various European honey bee subspecies (Smith and Brown, 1988). However, a clear distinction should be drawn between the identification of African mtDNA haplotypes and Africanized bees, particularly knowing that *A. m. scutellata* has been sporadically introduced into the United States before the arrival of the Africanized honey bees from South America in 1990 (Pinto et al., 2007). Africanization can occur either by gene flow from Africanized drones mating with European queens or by dispersal of Africanized queens in swarms (Smith and Brown, 1988). Thus, both European and *A. m. scutellata* haplotypes could carry Africanized traits, which renders the mtDNA a non-specific marker for this purpose. In our study, the African lineage represented 3.01% (n = 32) of the analyzed samples with four previously reported haplotypes (A1e, A1v, A4, A4p) and two novel haplotypes (A1-837-6-USA, A2-829-4-USA). The three highest percentages were found in three southern States: (LA: 25%, FL: 17.4%, TX: 12.5%), Figure 3. Both A1 and A4 are widely spread and common haplotypes of the African continent. They were identified at high frequencies in West and Central Africa among populations of *A. m. adansoni* and *A. m. jemenitica* (Dukku et al., 2022), in the Northeast of Africa (El-Niweiri and Moritz, 2008), among *A. m. intermissa* in North Africa (Achou M et al., 2015) as well as in the Middle East in the native range of *A. m. syriaca* (Alburaki et al., 2011). Meanwhile, Magnus et al.‘s study provided evidence of *A. m. syriaca* in the USA, which we did not identify in this current study (Magnus et al., 2014). According to the available records, this subspecies was imported to the USA in 1880 (Marcelino et al., 2022). *A. m. syriaca* is a subspecies of the African lineage A, which carries a distinct COI-COII profile compared to other subspecies of the A lineage (Alburaki et al., 2011). This unique mtDNA profile of *A. m. syriaca* (Z haplotypes)*,* which was confirmed *via* genomic analyses (Alburaki et al., 2013), allows reliable attribution of Z haplotypes to this specific subspecies (*A. m. syriaca*).

## 5 Conclusion

This study provides a comprehensive and unprecedented analysis of the mtDNA genetic diversity of the honey bee populations in the USA. It details and validates the methodology used to transit a widely used mtDNA genetic marker in honey bees from its traditional way to a more precise and systemic *in silico* test, eliminating discrepancies and reconciling haplotype identities between various investigations. This work identified and named fourteen novel haplotypes of three honey bee evolutionary lineages that have never been reported before, providing new insights into the evolutionary history of the USA’s honey bees since their importation to North America in the 1600s. Finally, from the perspective of national importance, the overwhelming reliance on two mtDNA haplotypes and a single lineage are all alarming signals of restrictions in the mtDNA haplotype diversity within the honey bee populations of the USA.

## Data Availability

The datasets presented in this study can be found in online repositories. The names of the repository/repositories and accession number(s) can be found in the article/Supplementary Material.
