# Supplementary material for: Honey bee populations of the USA display restrictions in their mtDNA haplotype diversity
Source: Front Genet. 2023 Jan 4;13:1092121. doi: 10.3389/fgene.2022.1092121 (PMC9845583; doi:10.3389/fgene.2022.1092121)
Supplement: Supplementary file 1 [file Table1.DOCX]

**Table S1.** Genetic diversity within the populations of the USA and its lineages. Haplotype diversity calculated based on endonuclease fragment variations (*H_1_*) ([Nei and Tajima 1981](#_ENREF_48)), Haplotype diversity computed on DNA sequences (*H_2_*), standard deviation of *H_2_* (sd), nucleotide diversity (π), Tajima’s statistic (*D*), Tajima’s P-values under normal (P_1_) and beta (P_2_) distributions. Level of significance: P < 0.05 (*), P < 0.01 (**), P < 0.001 (***).

| **Code** | ***H_1_*** | ***H_2_*** | $\boldsymbol{\pm}$ **sd** | **π** | ***D*** | **P_1_** | **P_2_** |
| --- | --- | --- | --- | --- | --- | --- | --- |
| AL | 0.706 | 0.766 | 0.005 | 0.005 | -5.76 | *** | *** |
| AK | 0.333 | 0.600 | 0.04 | 0.002 | -1.70 | 0.08 | *** |
| AZ | 0.508 | 0.604 | 0.002 | 0.003 | -10.34 | *** | *** |
| AR | 0.676 | 0.853 | 0.001 | 0.005 | -5.35 | *** | *** |
| CA | 0.565 | 0.584 | 0.001 | 0.004 | -10.33 | *** | *** |
| CO | 0.436 | 0.600 | 0.003 | 0.002 | 0.96 | 0.33 | 0.36 |
| CT | 0.467 | 0.589 | 0.005 | 0.002 | 0.86 | 0.38 | 0.41 |
| DE | 0.652 | 0.733 | 0.004 | 0.004 | -5.67 | *** | *** |
| DC | 0.628 | 0.714 | 0.006 | 0.002 | -1.12 | 0.26 | 0.31 |
| FL | 0.654 | 0.798 | 0.003 | 0.004 | -5.69 | *** | *** |
| GA | 0.640 | 0.717 | 0.004 | 0.004 | -6.36 | *** | *** |
| GU | 0.236 | 0.307 | 0.01 | 0.000 | -3.77 | *** | *** |
| HI | 0.595 | 0.595 | 0.01 | 0.005 | -7.48 | *** | *** |
| ID | 0.586 | 0.585 | 0.005 | 0.004 | -10.31 | *** | *** |
| IL | 0.608 | 0.801 | 0.003 | 0.003 | -0.78 | 0.43 | 0.55 |
| IN | 0.574 | 0.621 | 0.003 | 0.002 | -2.54 | * | *** |
| IA | 0.416 | 0.500 | 0.015 | 0.001 | -1.38 | 0.16 | *** |
| KS | 0.605 | 0.604 | 0.004 | 0.005 | -10.18 | *** | *** |
| KY | 0.529 | 0.557 | 0.008 | 0.003 | -9.47 | *** | *** |
| LA | 0.742 | 0.852 | 0.001 | 0.005 | -5.10 | *** | *** |
| ME | 0.554 | 0.699 | 0.003 | 0.003 | -7.42 | *** | *** |
| MD | 0.516 | 0.679 | 0.002 | 0.002 | -1.49 | 0.13 | *** |
| MA | 0.554 | 0.721 | 0.003 | 0.002 | -1.12 | 0.26 | 0.31 |
| MI | 0.685 | 0.753 | 0.005 | 0.004 | -5.78 | *** | *** |
| MN | 0.372 | 0.604 | 0.01 | 0.002 | -1.14 | 0.25 | 0.28 |
| MS | 0.492 | 0.492 | 0.002 | 0.002 | 1.09 | 0.27 | 0.29 |
| MO | 0.673 | 0.771 | 0.006 | 0.003 | -5.27 | *** | *** |
| MT | 0.612 | 0.695 | 0.004 | 0.003 | -1.89 | 0.05 | *** |
| NE | 0.344 | 0.692 | 0.004 | 0.002 | -1.49 | 0.13 | *** |
| NV | 0.614 | 0.680 | 0.004 | 0.004 | -7.36 | *** | *** |
| NJ | 0.598 | 0.655 | 0.004 | 0.004 | -7.36 | *** | *** |
| NM | 0.581 | 0.652 | 0.009 | 0.004 | -5.66 | *** | *** |
| NY | 0.638 | 0.637 | 0.003 | 0.005 | -7.44 | *** | *** |
| ND | 0.539 | 0.640 | 0.004 | 0.003 | -7.40 | *** | *** |
| OK | 0.605 | 0.652 | 0.004 | 0.004 | -6.70 | *** | *** |
| OR | 0.566 | 0.603 | 0.005 | 0.002 | -4.77 | *** | *** |
| PA | 0.577 | 0.577 | 0.008 | 0.003 | -10.16 | *** | *** |
| PR | 0.464 | 0.464 | 0.04 | 0.007 | -5.28 | *** | *** |
| SC | 0.584 | 0.718 | 0.003 | 0.003 | -6.408 | *** | *** |
| SD | 0.567 | 0.566 | 0.002 | 0.004 | -4.05 | *** | *** |
| TN | 0.654 | 0.782 | 0.005 | 0.004 | -7.40 | *** | *** |
| TX | 0.627 | 0.692 | 0.002 | 0.005 | -9.40 | *** | *** |
| UT | 0.489 | 0.510 | 0.009 | 0.003 | -9.40 | *** | *** |
| VT | 0.567 | 0.809 | 0.002 | 0.003 | -3.97 | *** | *** |
| VA | 0.763 | 0.790 | 0.001 | 0.005 | -6.26 | *** | *** |
| WA | 0.667 | 0.702 | 0.003 | 0.004 | -6.38 | *** | *** |
| WV | 0.598 | 0.688 | 0.004 | 0.002 | -1.12 | 0.26 | 0.31 |
| WI | 0.540 | 0.666 | 0.003 | 0.003 | -1.14 | 0.25 | 0.28 |
| **Lineage C** | **0.542** | **0.661** | **0.0008** | **0.002** | **-2.49** | ***** | *** |
| **Lineage M** | **0.720** | **0.793** | **0.002** | **0.002** | **-5.38** | *** | *** |
| **Lineage A** | **0.712** | **0.760** | **0.002** | **0.003** | **-6.17** | *** | *** |
| **National** | **0.597** | **0.701** | **0.0008** | **0.009** | **-2.66** | ****** | *** |
