# Supplementary figures and images for: Honey bee populations of the USA display restrictions in their mtDNA haplotype diversity
Source: Front Genet. 2023 Jan 4;13:1092121. doi: 10.3389/fgene.2022.1092121 (PMC9845583; doi:10.3389/fgene.2022.1092121)

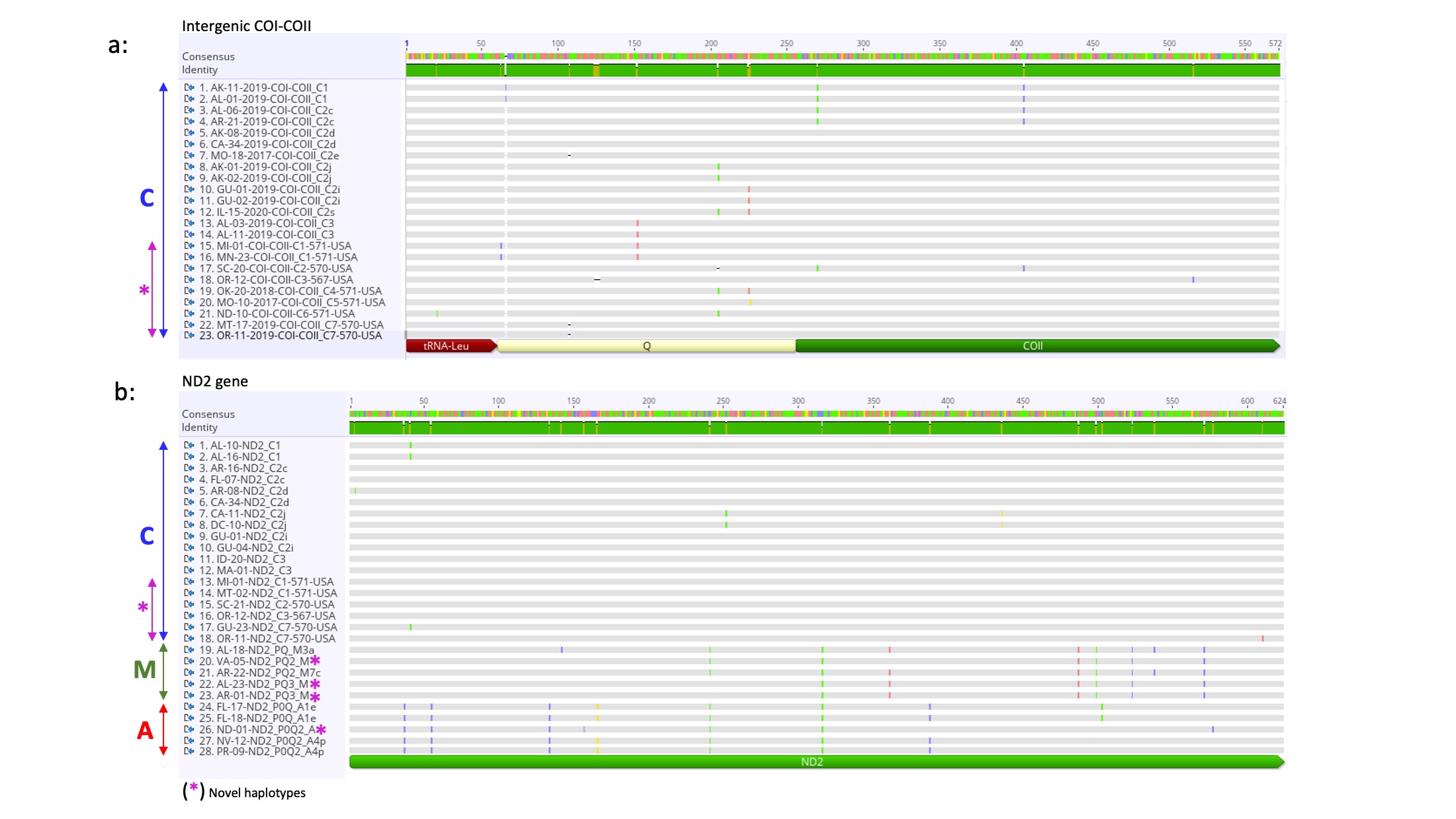

Supplement: Supplementary file 2 [file Image1.JPEG]
